# A microRNA Approach to Discriminate Cortical Low Bone Turnover in Renal Osteodystrophy

**DOI:** 10.1002/jbm4.10353

**Published:** 2020-03-25

**Authors:** Thomas L Nickolas, Neal Chen, Donald J McMahon, David Dempster, Hua Zhou, James Dominguez, Maria A Aponte, Joshua Sung, Pieter Evenepoel, Patrick C D'Haese, Fabrice Mac‐Way, Rosa Moyses, Sharon Moe

**Affiliations:** ^1^ Department of Medicine Columbia University Medical Center New York NY USA; ^2^ Division of Nephrology Indiana University School of Medicine Indianapolis IN USA; ^3^ Department of Pathology and Cell Biology Columbia University New York NY USA; ^4^ Regional Bone Center Helen Hayes Hospital New York NY USA; ^5^ Department of Microbiology and Immunology, Laboratory of Nephrology Katholieke Universiteit Leuven, University of Leuven Leuven Belgium; ^6^ Department of Biomedical Sciences, Laboratory of Pathophysiology Antwerp University Wilrijk Belgium; ^7^ CHU de Québec Research Center, L'Hôtel‐Dieu de Québec Hospital, Endocrinology and Nephrology Axis, Faculty and Department of Medicine Université Laval Quebec City Canada; ^8^ Laboratório de Investigação Médica 16 Hospital das Clinicas da Faculdade de Medicina da Universidade de Sao Paulo Sao Paulo Brazil; ^9^ Department of Medicine Roudebush Veterans Administration Medical Center Indianapolis IN USA

**Keywords:** microRNA, RENAL OSTEODYSTROPHY

## Abstract

A main obstacle to diagnose and manage renal osteodystrophy (ROD) is the identification of intracortical bone turnover type (low, normal, high). The gold standard, tetracycline‐labeled transiliac crest bone biopsy, is impractical to obtain in most patients. The Kidney Disease Improving Global Outcomes Guidelines recommend PTH and bone‐specific alkaline phosphatase (BSAP) for the diagnosis of turnover type. However, PTH and BSAP have insufficient diagnostic accuracy to differentiate low from non‐low turnover and were validated for trabecular turnover. We hypothesized that four circulating microRNAs (miRNAs) that regulate osteoblast (miRNA‐30b, 30c, 125b) and osteoclast development (miRNA‐155) would provide superior discrimination of low from non‐low turnover than biomarkers in clinical use. In 23 patients with CKD 3‐5D, we obtained tetracycline‐labeled transiliac crest bone biopsy and measured circulating levels of intact PTH, BSAP, and miRNA‐30b, 30c, 125b, and 155. Spearman correlations assessed relationships between miRNAs and histomorphometry and PTH and BSAP. Diagnostic test characteristics for discriminating low from non‐low intracortical turnover were determined by receiver operator curve analysis; areas under the curve (AUC) were compared by χ^2^ test. In CKD rat models of low and high turnover ROD, we performed histomorphometry and determined the expression of bone tissue miRNAs. Circulating miRNAs moderately correlated with bone formation rate and adjusted apposition rate at the endo‐ and intracortical envelopes (ρ = 0.43 to 0.51; *p* < 0.05). Discrimination of low versus non‐low turnover was 0.866, 0.813, 0.813, and 0.723 for miRNA‐30b, 30c, 125b, and 155, respectively, and 0.509 and 0.589 for PTH and BSAP, respectively. For all four miRNAs combined, the AUC was 0.929, which was superior to that of PTH and BSAP alone and together (*p* < 0.05). In CKD rats, bone tissue levels of the four miRNAs reflected the findings in human serum. These data suggest that a panel of circulating miRNAs provide accurate noninvasive identification of bone turnover in ROD. © 2020 The Authors. *JBMR Plus* published by Wiley Periodicals, Inc. on behalf of American Society for Bone and Mineral Research.

## Introduction

Renal osteodystrophy (ROD) is a progressive disease of cortical bone.^1^, ^2^, ^3^, ^4^, ^5^ In ROD, cortical density, geometry, microarchitecture, and strength undergo progressive deterioration caused by the combined actions of high circulating levels of PTH and elevated bone remodeling rates.^1^, ^2^, ^6^ In contrast, ROD is associated with trabecular hypertrophy rather than the trabecular dropout and disconnectivity that is associated with postmenopausal and glucocorticoid‐induced osteoporosis.^2^ Therefore, CKD patients are at increased risk of cortical‐type bone fractures; since 1992 there has been a doubling of peripheral fracture incidence in patients with end‐stage kidney disease on dialysis.^7^, ^8^

Although cortical bone is critical to the pathogenesis of ROD, trabecular rather than cortical remodeling rates are used to determine ROD type and to inform ROD treatment decisions.^3^, ^9^, ^10^, ^11^, ^12^ Indeed, the Kidney Disease Improving Global Outcomes (KDIGO) Guidelines defined ROD by bone turnover, mineralization, and volume in trabecular bone based on quantitative histomorphometry of tetracycline double‐labeled transiliac crest bone biopsy.^9^ Furthermore, the primary goal of ROD treatment is to reduce high bone turnover with calcitriol and its analogues and/or calcimimetics, at the same time as avoiding the development of low turnover through excessive use of these same agents. Because widespread use of bone biopsy in the clinic for either diagnosis or treatment monitoring of ROD is impractical, KDIGO recommended that clinical use (ie, starting/stopping) of agents used to treat ROD are guided by the biomarkers PTH and bone‐specific alkaline phosphatase (BSAP) based on their ability to discriminate low turnover in trabecular bone.^13^ However, large‐scale multinational bone biopsy studies in dialysis patients demonstrated that PTH and BSAP were poor guides for ROD treatment because of their suboptimal discrimination for low turnover ROD (areas under the curve [AUCs] 0.701 and 0.757, respectively).^3^, ^10^ Although we assume that relationships between the cortical, endocortical, and trabecular bone compartments and bone turnover, bone turnover markers (BTMs), and ROD treatments are similar, there are no comparative studies of these relationships. Thus, there is an unmet clinical need to identify noninvasive biomarkers with strong diagnostic accuracy allowing differentiation between low from non‐low turnover ROD; it is not clear whether the development and study of novel biomarkers of turnover should measure cortical rather than trabecular turnover.

MicroRNAs (miRNAs) are small noncoding sequences of approximately 22 nucleotides that bind to the 3′‐untranslated regions of mRNAs to alter gene expression by inhibiting translation or promoting degradation of target mRNAs. Experimental studies have examined miRNA expression during osteoblast and osteoclast development^14^, ^15^, ^16^: Bone cell phenotypic effects of miRNA substitutions and knockdowns have been described^17^, ^18^ and the impact of hormones and RANK^19^ on miRNA expression signatures. In non‐CKD patients with osteoporosis, relationships between miRNAs and histomorphometry have been reported,^20^ and dysregulation in levels of circulating miRNA expression has been associated with osteoporosis^21^, ^22^, ^23^ and fractures.^24^, ^25^ In CKD patients, levels of miRNAs and PTH have been correlated^26^; in cell culture, inorganic phosphate was shown to modulate osteoclastogenesis by miRNA‐233.^27^ miRNAs have not been tested as biomarkers of turnover in CKD. We hypothesized that (i) circulating miRNAs reported in previous investigations to regulate osteoblast (miRNA‐30b, 30c, 125b^15^, ^28^, ^29^, ^30^) and osteoclast (miRNA‐155^31^, ^32^) development are associated with low turnover in all bone compartments; (ii) PTH, BSAP, and circulating BTMs used in clinical practice reflect turnover within cortical and endocortical bone; and (iii) the turnover within all three bone compartments are highly correlated. We also hypothesized that the circulating miRNA profile of low turnover ROD detected in humans will be reflected at the bone tissue level in a rat model of CKD with low turnover ROD.

## Subjects and Methods

### Cohort

The Institutional Review Board of Columbia University Irving Medical Center (CUIMC) approved this cross‐sectional study; all subjects provided written informed consent. The study design has been previously described.^1^, ^33^, ^34^ In brief, 23 patients with CKD stages 3 to 5D were recruited from the general nephrology clinics of CUIMC. The estimated glomerular filtration rate (eGFR) was determined by the Modification of Diet in Renal Disease short formula for CKD patients not on dialysis.^35^ Patients were excluded if they had a history of malignancy or bilateral lower extremity amputations; were nonambulatory; were institutionalized; or used bisphosphonates, teriparatide, gonadal steroids, aromatase inhibitors, or anticonvulsants that induce cytochrome‐P450. All CKD etiologies were eligible. Thirteen participants had a history of fracture: five participants had vertebral fractures (occult and clinical); four participants had an ankle or metatarsal fracture; four participants had a radius fracture; one patient had a hip, clavicle, rib, or pelvic fracture; and eight participants had multiple fractures. One participant had two fractures that occurred within 12 months of bone biopsy and measurement of miRNAs and BTMs. In sensitivity analysis, removal of this participant from analysis did not materially change the results; thus, this participant was included in this research.

### Laboratory measurements and circulating microRNA isolation and analysis

Fasting blood samples were obtained in the morning. Routine laboratories were measured by Quest Diagnostics (Secaucus, NJ, USA). PTH and BSAP were measured at CUIMC in a research laboratory. Calciotropic hormones and BTMs were measured at CUIMC in a specialized research laboratory. Intact PTH, serum total 25‐hydroxyvitamin D (25‐OHD), BSAP, N‐Mid osteocalcin (OCN), P1NP, tartrate‐resistant acid phosphatase 5b (TRAP‐5b), and CTx were measured by Roche Elecsys 2010 Analyzer (Roche Diagnostics, Indianapolis, IN, USA). C‐terminal fibroblast growth factor 23 (FGF‐23) and sclerostin (SOST) were measured by ELISA (Immunotopics, San Clemente, CA, USA) and TECOmedical (Sissach, Switzerland), respectively. Intra‐ and interassay precisions are intact PTH 1.0% and 4.4%; BSAP 6.0% and 8.0%; OCN 0.8% and 2.9%; P1NP 1.1% and 5.5%; CTx 1.1% and 5.5%, FGF‐23 2.40% and 4.70%, and SOST 3.1% and 3.5%, respectively. For 25‐OHD the normal range is >30 ng/mL and the interassay precision is 2.6% to 4.4%. miRNA was measured at Indiana University School of Medicine: total RNA was isolated from serum and miRNA expression determined by real‐time PCR using TaqMan miRNA assay (Applied Biosystem, Foster City, CA, USA) normalized by spiking with *C. elegans* miRNA‐39.^36^

### Transiliac bone biopsy and histomorphometry

After double‐labeling with tetracycline in a 3‐:12‐:3‐day sequence, transiliac bone biopsy was performed using a 7.5‐mm Bordier‐type trephine. Specimens were fixed in 70% ethanol, processed without decalcification, and embedded in methylmethacrylate. Histomorphometry was performed on Goldner's trichrome stained or unstained sections with a morphometric program (OsteoMeasure, Version 4.00C; OsteoMetrics, Inc., Atlanta, GA, USA). The trabecular, endocortical, and cortical bone compartments were delineated manually prior to measurement of histomorphometric parameters (Supplemental Fig. 1). All variables were expressed and calculated according to the recommendations of the ASBMR for the trabecular, endocortical, and cortical bone compartments.^37^ Classification of ROD was assessed by interpreting histology and histomorphometry indices according to the Turnover, Mineralization, and Volume (TMV) system.^38^

### Animal models

The Cy/+ rat model of CKD was used to assess bone expression of miRNAs. Cy/+ rats are characterized by an autosomal dominant progressive cystic kidney disease that is not allogenic with human ADPKD.^39^ In this rat model, chronic kidney disease‐mineral and bone disorder (CKD‐MBD) develops spontaneously, with a much faster progression to end‐stage disease in male animals by 30 to 35 weeks of age, whereas female rats do not develop azotemia even as old as 21 months,^40^ or after oophorectomy (unpublished data). The Cy/+_IU_ colony of rats has been bred at Indiana University for nearly 20 years. The model recapitulates CKD‐MBD with progressive kidney disease, hyperphosphatemia, secondary hyperparathyroidism, elevated FGF‐23, resulting in ROD and vascular calcification. Importantly, the slowly progressive nature of the model allows for examination of interventions that differentially affect bone remodeling. Specifically, we have induced low turnover bone remodeling by two methods: with calcium in the drinking water (calcium binders) and zoledronic acid.^41^, ^42^ In brief, CKD animals (*n* = 8 to 10 each group) began treatment at 25 weeks for a total of 10 weeks and received: (i) no treatment (control CKD = high PTH/ high turnover; (ii) 3% calcium in the drinking water (CKD/Ca group = low PTH/low turnover); or (iii) a single injection of 20 μg/kg of zoledronic acid (CKD/Zol group = high PTH/low turnover). At 35 weeks of age, animals were euthanized and bone tissue was collected. Bone histomorphometry was performed as previously reported.^43^ RNA was isolated from tibia, and bone miRNA expression was determined by real‐time PCR using TaqMan miRNA assay as described above. All procedures were reviewed and approved by the Indiana University School of Medicine Institutional Animal Care and Use Committee.

### Statistical methods

For human subjects, statistical analyses were conducted using SAS (version 9.4; SAS Institute, Cary, NC, USA). Continuous data were evaluated for normality before statistical testing and log‐transformed when appropriate. Relationships between miRNAs, PTH, BSAP, BTMs, and histomorphometric parameters (bone formation rate / bone surface [BFR/BS]; adjusted apposition rate [AjAR]; mineralization lag time [MLT]) were determined by Spearman correlations at the trabecular, endocortical, and intracortical bone compartments. The cohort was stratified into patients with low and non‐low turnover based on the BFR/BS at the intracortical envelope because of the known importance of cortical bone in the pathogenesis of impaired bone quality in patients with CKD.^1^ The lowest tertile of intracortical BFR/BS defined low turnover because there are no normative reference data for cortical bone. Group differences for continuous parameters between patients with low versus non‐low turnover were determined by Wilcoxon rank sum. Standard receiver operator characteristic (ROC) curve analysis was performed to determine the ability of biomarkers to discriminate between low and non‐low turnover. We also created two biomarker panels for ROC analyses: (i) an miRNA panel including all four miRNAs; and (ii) a CKD‐MBD panel including BSAP and CTX. Rat bone miRNA expression was analyzed using one‐way ANOVA and within group comparisons by Fisher's post hoc analysis. The results are expressed as means ± SD, with *p* < 0.05 considered significant (GraphPad Prism Software; GraphPad, La Jolla, CA, USA).

## Results

### Cohort characteristics, levels of circulating biomarkers, and relationships with kidney function

Cohort characteristics stratified by low and non‐low turnover in intracortical bone are presented in Table 1. In patients with low intracortical turnover, intracortical BFR/BS and mineral apposition rate were lower whereas MLT was higher. In contrast, among patients with low turnover based on intracortical remodeling, only BFR/BS was significantly lower in the trabecular and endocortical compartments. Bone turnover groups did not differ by demographics, kidney function, or comorbid status. Biochemical markers of CKD‐MBD (calcium, phosphorus, 25(OH)D, PTH, and FGF‐23), bone formation (BSAP, OCN, P1NP) and resorption (C‐telopeptide, TRAP5B) markers, and SOST did not differ between low and non‐low turnover. In contrast, circulating levels of miRNA‐30b, 30c, and 125b were significantly lower in subjects with low compared with non‐low turnover. Levels of BSAP, P1NP, and TRAP‐5b, and circulating miRNAs were not affected by eGFR or dialysis status (Table 2 and Supplemental Fig. 2). In contrast, levels of PTH, vitamin D, OCN, CTx, SOST, and FGF‐23 were related to kidney function (data not shown).

**Table 1:** Cohort Characteristics by Bone Turnover Level Status

	Bone turnover group		
*N* = 23	Low bone turnover (*n* = 7)	Non‐low bone turnover (*n* = 16)	*p* Value
Histomorphometry–median (IQR)			
Intracortical bone formation rate / bone surface (mm^3^/mm^2^/year)	0.0017 (0.0005; 0.0063)	0.0181 (0.0133; 0.0460)	0.0005
Intracortical mineral apposition rate (μm/d)	0.3000 (0.3000; 0.3000)	0.6637 (0.5248; 0.7240)	0.0008
Intracortical mineralization lag time (days)	48.2 (34.2; 165.9)	18.8 (12.8; 31.1)	0.02
Trabecular bone formation rate / bone surface (mm^3^/mm^2^/year)	0.0024 (0.0001; 0.0060)	0.0101 (0.0059; 0.0209)	0.02
Trabecular mineral apposition rate (μm/d)	0.3000 (0.3000; 0.6699)	0.5296 (0.5087; 0.6494)	NS
Trabecular mineralization lag time (days)	47.0 (18.5; 1067.8)	33.4 (22.9; 61.0)	NS
Endocortical bone formation rate / bone surface (mm^3^/mm^2^/year)	0.0051 (0.0006; 0.0115)	0.0156 (0.0099; 0.0249)	0.02
Endocortical mineral apposition rate (μm/d)	0.3000 (0.3000; 0.6021)	0.5323 (0.4224; 0.5694)	NS
Endocortical mineralization lag time (days)	32.7 (3.9; 106.3)	26.0 (16.0; 84.7)	NS
Demographics			
Age–mean years (SD)	71 (9)	63 (14)	NS
Female–*N* (%)	4 (57%)	11 (69%)	NS
White–*N* (%)	4 (57%)	10 (63%)	NS
Comorbids–*N* (%)			
Prevalent fracture	1 (14%)	12 (75%)	NS
Dialysis	1 (14%)	5 (31%)	NS
History of transplant	0 (0%)	1 (11%)	NS
Biochemical–median (IQR)			
GFR (mL/min/1.74)	22 (17; 57)	33 (14; 36)	NS
Calcium (mg/dL)	9.6 (9.5; 10.0)	9.3 (9.0; 10.1)	NS
Phosphorus (mg/dL)	3.9 (3.5; 6.0)	3.8 (3.2; 4.3)	NS
25‐hydroxyvitamin D (ng/mL)	38.5 (24.8; 46.1)	25.8 (15.0; 44.5)	NS
PTH (pg/mL)	84 (54; 149)	78 (34; 224)	NS
Bone‐specific alkaline phosphatase (U/L)	29 (23; 38)	36 (23; 56)	NS
Osteocalcin (ng/mL)	45 (26; 155)	71 (33; 171)	NS
P1NP (μL/L)	76 (47; 233)	138 (79; 213)	NS
C‐Telopeptide (ng/mL)	0.723 (0.428; 1.570)	0.847 (0.563; 1.345)	NS
TRAP‐5b (U/L)	2.839 (2.549; 3.336)	4.275 (2.906; 5.860)	NS
Sclerostin (ng/mL)	1.592 (0.933; 1.991)	1.545 (1.083; 1.973)	NS
Fibroblast growth factor 23 (RU/mL)	163 (95; 362)	162 (128; 515)	NS
miRNA–median (IQR)			
miR‐30b	0.774 (0.421; 1.447)*	2.341 (1.205; 3.562)	0.007
miR‐30c	0.848 (0.500; 1.636)*	2.036 (1.328; 3.409)	0.02
miR‐125b	2.989 (2.407; 4.202)*	9.188 (3.386; 11.416)	0.02
miR‐155	0.802 (0.673; 1.159)	1.218 (0.861; 1.457)	0.1

GFR = glomerular filtration rate; IQR = interquartile range; NS = not significant; TRAP‐5b = tartrate‐resistant acid phosphatase 5b.

* *p* < 0.05 low versus non‐low.

**Table 2:** Spearman Correlations Between miRNAs and Biomarkers of CKD‐MBD and Bone Turnover

	miRNA‐30c	miRNA‐125b	miRNA‐155	Kidney function	Calcium	Phosphorus	25(OH)D	Intact PTH	BSAP	Osteocalcin	P1NP	C‐Telopeptide	TRAP‐5b	Sclerostin	FGF‐23
miRNA‐30b	**0.98**	**0.77**	**0.51**	0.28	**−0.41**	**−0.54**	−0.15	−0.12	−0.05	−0.06	0.02	−0.09	0.05	−0.25	−0.15
*p* Value	**<.0001**	**<.0001**	**.01**	.2	**.05**	**.007**	.5	.6	.8	.8	.9	.7	.8	.3	.6
miRNA‐30c		**0.75**	**0.57**	0.32	−**0.44**	−**0.54**	−0.15	−0.12	−0.03	−0.11	−0.01	−0.13	0.01	−0.25	−0.15
*p* Value		**<.0001**	**0.005**	0.1	**0.03**	**0.007**	0.5	0.6	0.9	0.7	1.0	0.5	1.0	0.3	0.6
miRNA‐125b			**0.39**	0.21	−0.25	−**0.49**	−0.04	−0.08	−0.01	0.11	0.11	0.04	0.24	−0.32	−0.19
*p* Value			**0.066**	0.3	0.3	**0.02**	0.8	0.7	0.9	0.6	0.6	0.9	0.3	0.2	0.5
miRNA‐155				0.17	−0.29	−0.17	−0.35	−0.14	−0.30	−0.23	0.06	−0.32	0.04	0.06	0.23
*p* Value				0.4	0.2	0.4	0.1	0.5	0.2	0.3	0.8	0.1	0.9	0.8	0.4

BSAP = bone‐specific alkaline phosphatase; CKD‐MBD = chronic kidney disease‐mineral and bone disorder; FGF‐23 = fibroblast growth factor 23; miRNA = microRNA; TRAP‐5b = tartrate‐resistant acid phosphatase 5b. Boldface indicates significant correlations.

### Relationships between histomorphometry, miRNAs, biochemical makers of CKD‐MBD, and bone turnover

Spearman correlations were used to evaluate relationships between histomorphometric parameters in the trabecular, endocortical, and intracortical compartments and miRNAs and biomarkers of CKD‐MBD and BTMs (Tables 2 and 3). BFR/BS was correlated moderately to strongly between compartments: Although trabecular BFR/BS described 72% of the heterogeneity in endocortical BFR/BS, it described only 59% of the heterogeneity in intracortical BFR/BS. CKD‐MBD biomarkers, BTMs, and miRNAs were moderately related to formation and mineralization measures at the trabecular, endocortical, and intracortical regions. For CKD‐MBD biomarkers, PTH and 25(OH)D were directly related to BFR/BS in trabecular bone and BSAP was directly related to BFR/BS in trabecular and intracortical bone. For BTMs, OCN and CTx were directly related to BFR/BS in all bone compartments. For the miRNAs, miRNA‐30b, 30c, and 125b were directly and strongly related to each other and were positively and moderately related to miRNA‐155. miRNA‐30b, 30c, and 125b were inversely related to phosphorus levels; miRNA‐30b and 30c were inversely related to calcium. None of the miRNAs were related to CKD‐MBD biomarkers or BTMs. miRNA‐30b, 30c, and 125b were moderately and directly related to the AjAR in intracortical bone and 125b was inversely related to MLT in intracortical bone.

**Table 3:** Spearman Correlations Between Dynamic Histomorphometry, miRNAs, PTH, BSAP, and Markers of Bone Turnover

	BFR/BS	AjAR	MLT
Trabecular bone			
Endocortical bone	**0.85** c	0.34	**0.57** b
Cortical bone	**0.77** c	**0.70** c	0.33
miRNA‐30b	0.16	0.25	−0.20
miRNA‐30c	0.09	0.20	−0.17
miRNA‐125b	0.09	0.18	−0.14
miRNA‐155	−0.18	−0.15	0.06
Intact PTH	**0.54** b	0.08	0.11
25(OH)D	**−0.45** a	−0.21	0.10
BSAP	**0.64** c	0.36	−0.21
Osteocalcin	**0.55** a	0.27	−0.11
P1NP	**0.49** a	0.37	−0.32
C‐Telopeptide	**0.66** c	0.27	−0.13
TRAP‐5b	**0.44** a	0.25	−0.15
Sclerostin	0.05	−0.22	0.20
Fibroblast growth factor 23	0.01	−0.28	0.34
Endocortical bone			
Cortical bone	**0.74** c	**0.60** b	**0.48** a
miRNA‐30b	0.27	**0.44** a	−0.23
miRNA‐30c	0.15	0.40	−0.22
miRNA‐125b	0.12	0.16	−0.10
miRNA‐155	−0.02	0.15	−0.05
Intact PTH	0.23	−0.32	0.35
25(OH)D	−0.38	−0.13	0.09
BSAP	0.34	0.00	−0.07
Osteocalcin	**0.47** a	0.06	−0.01
P1NP	**0.49** a	0.32	−0.22
C‐Telopeptide	**0.44** a	−0.09	0.29
TRAP‐5b	0.32	0.05	0.12
Sclerostin	0.19	−0.07	−0.02
Fibroblast growth factor 23	0.06	−0.17	−0.08
Cortical bone			
miRNA‐30b	0.35	**0.51** a	−0.31
miRNA‐30c	0.27	**0.42** a	−0.24
miRNA‐125b	0.34	**0.52** a	**−0.50** a
miRNA‐155	0.01	0.13	0.01
Intact PTH	0.20	0.03	0.03
25(OH) D	−0.22	−0.12	−0.03
BSAP	**0.48** a	0.24	−0.30
Osteocalcin	**0.49** a	0.29	−0.24
P1NP	0.43	0.19	−0.13
C‐Telopeptide	**0.42** a	0.20	−0.04
TRAP‐5b	0.43	0.30	−0.15
Sclerostin	−0.06	−0.15	0.09
Fibroblast growth factor 23	−0.07	−0.21	0.03

AjAR = adjusted apposition rate; BFR/BS = bone formation rate / bone surface; BSAP = bone‐specific alkaline phosphatase; CKD‐MBD = chronic kidney disease‐mineral and bone disorder; miRNA = microRNA; MLT = mineralization lag time; TRAP‐5b = tartrate‐resistant acid phosphatase 5b. Boldface indicates significant correlations.

a *p* < 0.05.

b *p* < 0.01.

c *p* < 0.001.

We used discrimination analysis to determine and compare diagnostic test characteristics of miRNAs, markers of CKD‐MBD (PTH, BSAP), and BTMs to differentiate low from non‐low turnover in all bone compartments (Table 4). In trabecular bone, markers of CKD‐MBD and BTMs moderately discriminated low turnover. A CKD‐MBD biomarker panel, including BSAP and CTx, had good discrimination for low turnover (AUC 0.882; 95% CI, 0.731 to 1.000) that was superior to the individual miRNAs, but not to the miRNA panel. In endocortical bone, none of the individual biomarkers discriminated low turnover; however, the miRNA panel of all four miRNAs had excellent discrimination (AUC 0.982; 95% CI, 0.940 to 1.000) that was superior to the other individual BTM and the CKD‐MBD panel. In intracortical bone, none of the markers of CKD‐MBD or BTM discriminated, but all miRNAs moderately discriminated low turnover. The miRNA panel highly discriminated low turnover (AUC 0.929; 95% CI, 0.821 to 1.000), which was superior to other biomarkers.

**Table 4:** Discrimination of Low Turnover at the Trabecular, Endocortical, and Cortical Bone Compartments for Biomarkers of CKD‐MBD, Bone Turnover, and miRNAs

Trabecular^a^				
	AUC	95% CI	*p* Value versus BTM panel	*p* Value versus miRNA panel
PTH	0.843	0.666 to 1.000	0.7	0.2
BSAP	0.779	0.570 to 1.000	0.3	0.4
CTx	0.873	0.714 to 1.000	0.7	0.1
miR‐30b	0.539	0.264 to 0.814	0.02	0.6
miR‐30c	0.569	0.301 to 0.836	0.03	0.7
miR‐125b	0.490	0.250 to 0.731	0.01	0.4
miR‐155	0.520	0.241 to 0.798	0.01	0.5
BTM panel	0.882	0.731 to 1.000	–	0.1
miRNA panel	0.673	0.339 to 1.000	0.1	–

Endocortical^a^				
	AUC	95% CI	*p* Value versus BTM panel	*p* Value versus miRNA panel
PTH	0.554	0.289 to 0.817	0.2	0.002
BSAP	0.553	0.311 to 0.797	0.3	0.0004
CTx	0.661	0.400 to 0.922	0.5	0.02
miR‐30b	0.625	0.388 to 0.863	0.8	0.004
miR‐30c	0.554	0.297 to 0.810	0.5	0.001
miR‐125b	0.527	0.278 to 0.775	0.4	0.0003
miR‐155	0.580	0.295 to 0.865	0.6	0.004
BTM panel	0.670	0.411 to 0.928	‐	0.03
miRNA panel	0.982	0.940 to 1.000	0.03	‐

Intracortical^a^				
	AUC	95% CI	*p* Value versus BTM panel	*p* Value versus miRNA panel
PTH	0.509	0.251 to 0.766	0.5	0.006
BSAP	0.589	0.349 to 0.830	0.5	0.02
CTx	0.576	0.289 to 0.863	0.9	0.04
miR‐30b	0.866	0.709 to 1.000	0.1	0.4
miR‐30c	0.813	0.610 to 1.000	0.3	0.2
miR‐125b	0.813	0.635 to 0.990	0.2	0.2
miR‐155	0.723	0.499 to 0.948	0.5	0.06
BTM panel	0.598	0.359 to 0.837	‐	0.02
miRNA panel	0.929	0.821 to 1.000	0.02	‐

AUC = area under the curve; BSAP = bone‐specific alkaline phosphatase; BTM = bone turnover marker; CKD‐MBD = chronic kidney disease‐mineral and bone disorder; miRNA = microRNA.

a Low turnover in each compartment was based on the lowest compartmental tertile of bone formation rate / bone surface.

### Bone tissue miRNA expression in CKD rats with low and high turnover ROD

**Figure 1:**
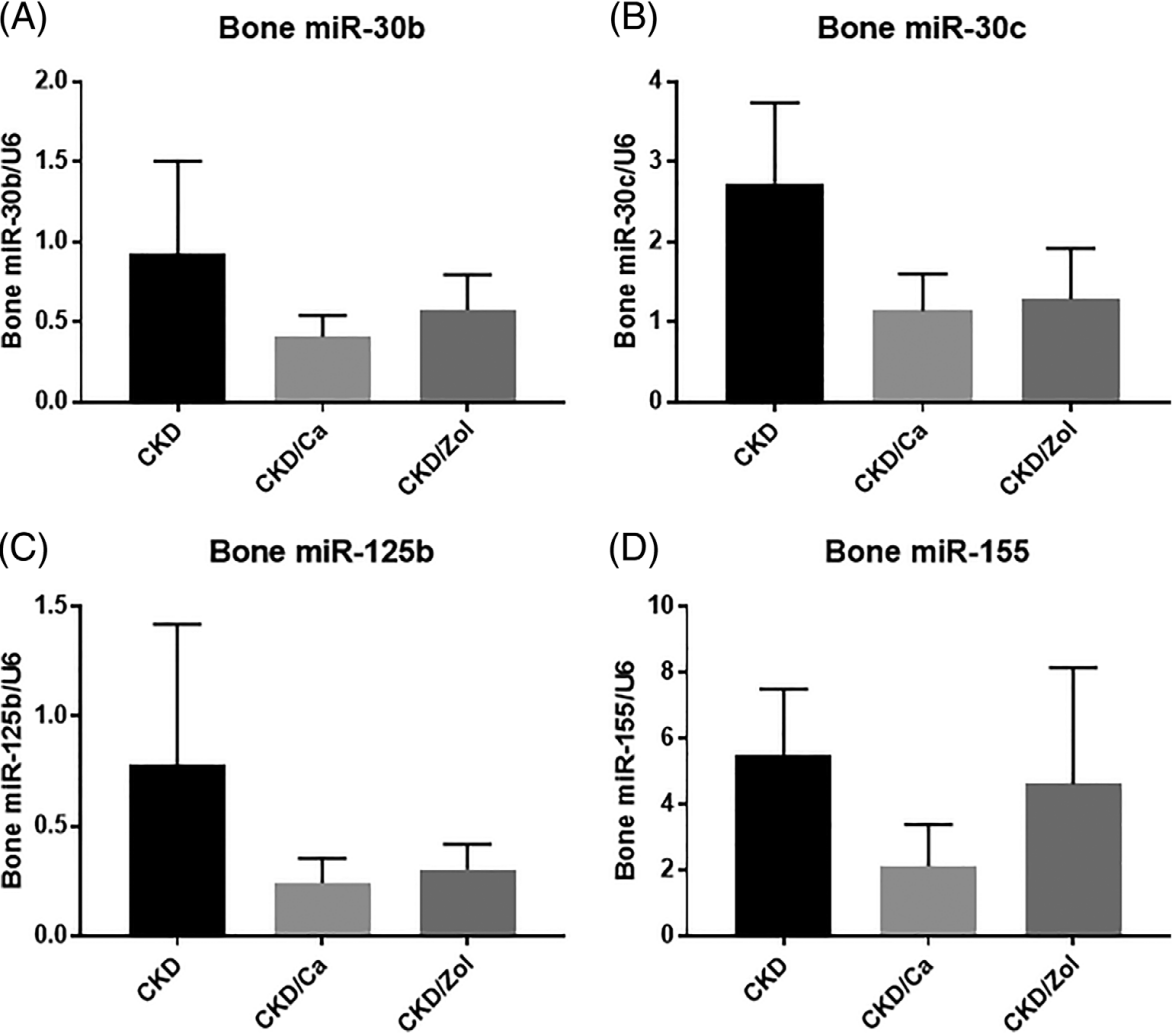
Quantification of miRNA‐30b (A), 30c (B), 125b (C), and 155 (D) expression in bone tissue from rats with high and low turnover renal osteodystrophy. Data are shown as mean ± SD (*n* = 8 to 10 rats each group). *p < 0.05 CKD versus CKD/Ca or CKD/Zol.

We induced low bone turnover in CKD rats by either adding calcium (3%) in the drinking water (low turnover, low PTH) or administration of a single dose of zoledronic acid (low turnover, high PTH), whereas CKD rats without treatment had high turnover and high PTH. Histomorphometric analysis of bone tissue confirmed the type of turnover induced by each intervention (Supplemental Fig. 3). We also quantified bone tissue expression of the miRNA 30b, 30c, 125b, and 155 in the CKD rats (Fig. 1). Levels of all four miRNAs were lower in rats with low turnover and low PTH compared with rats with high turnover. In rats with low bone turnover and high PTH, levels of miRNA‐30b, 30c, and 125b, but not 155 were lower compared with rats with high turnover. Levels of miRNAs did not differ between rats with low bone turnover induced by dietary calcium or zoledronic acid.

## Discussion

We report relationships between bone turnover in the trabecular, endocortical, and intracortical compartments and both traditional and novel circulating markers of bone turnover. Contrary to our hypothesis, differences in bone turnover rates were present between the bone compartments, and turnover in the trabecular and intracortical compartments was similar only 60% of the time. Although we also hypothesized that discrimination of low turnover by markers of CKD‐MBD, BTMs, and miRNAs would be similar within the three bone compartments, we found differences: Markers of CKD‐MBD and BTMs discriminated low turnover only in trabecular bone and miRNAs discriminated low turnover only in cortical bone. We used combinations of biomarkers to determine if discrimination could be significantly enhanced in comparison to the individual biomarkers; we found that a CKD‐MBD panel (BSAP, CTx) had highest discrimination in trabecular bone and that a miRNA panel had highest discrimination in endocortical and intracortical bone. Furthermore, we demonstrated that the circulating miRNA profile for low turnover ROD in humans was mimicked at the bone tissue level in two rat models of low bone turnover: PTH lowering therapy with calcium or by an antiresorptive agent.

Cortical bone is critically important to the pathogenesis of ROD and CKD‐associated fractures. Cortical bone comprises more than 75% of the skeleton and is a critical component of bone strength. Indeed, reductions in cortical thickness were shown to have a greater negative impact on whole bone strength than reductions in either trabecular number or thickness,^44^ and small increases in cortical porosity disproportionately affect bone strength and may contribute substantially to the risk of fractures.^45^, ^46^ ROD impairs cortical density, geometry, and microarchitecture based on the actions of hyperparathyroidism and elevated bone remodeling rates.^1^, ^2^, ^3^, ^4^, ^5^ In a longitudinal study of 53 patients with CKD 2‐5D, Nickolas and colleagues^1^ used HR‐pQCT to assess the effects of kidney disease on the skeleton. They reported that (i) cortical density and thickness decreased by 1.3% and 2.8% per year, respectively; (ii) cortical porosity increased by 4.2% per year; and (iii) trabecular microarchitecture was unchanged. They also reported that the cortical changes were driven by both elevated levels of PTH and bone turnover as measured by BTMs. Sharma and colleagues^6^ performed transiliac crest bone biopsy in 14 patients with CKD 5‐5D and quantified defects in the trabecular and cortical compartments by μCT. Although trabecular microarchitecture was relatively preserved, cortices were thinned and porous in all patients. Cortical defects were related to higher levels of PTH. The clinical relevance of cortical defects in ROD is manifested by the higher incidence of peripheral compared with central fractures.^7^ Whereas evidence for the importance of cortical bone in the pathophysiology of ROD and CKD‐associated fractures is well‐established, the assessment of ROD‐type by markers of CKD‐MBD and BTMs is based on relationships within trabecular bone, under the assumption that turnover in all bone compartments are highly correlated and because trabecular bone is assumed to be the most metabolically active bone compartment. For the first time in CKD patients, we report on comparisons between bone‐biopsy‐derived compartmental turnover and markers of CKD‐MBD, BTMs, and novel miRNA panel. Our findings highlight differences in turnover between compartments that may be relevant to ROD diagnosis and management.

Tetracycline double‐labeled iliac crest bone biopsy is the gold standard method to determine ROD turnover type. However, bone biopsy is not practical to obtain in the vast majority of CKD patients. Therefore, KDIGO recommended using PTH and BSAP both to define turnover‐type and to inform the treatment of ROD. Defining turnover type, especially discriminating low from non‐low turnover, is critical to managing ROD.^47^ Currently accepted treatment strategies for ROD include the use of vitamin D analogs and/or calcimimetics to suppress or mitigate the increase in PTH that occurs with declining kidney function. Another critical reason to define turnover type in ROD is to avoid treatment‐induced oversuppression of bone remodeling, as low turnover ROD has been associated with increased risk of fractures and vascular calcifications.^48^, ^49^, ^50^ Furthermore, recent updates to the 2017 KDIGO Guidelines on the treatment of osteoporosis in patients with CKD recommend defining turnover type before starting antiosteoporosis medications so that these agents are not given to patients with low turnover.^47^ A major limitation of this approach is the insufficient adequacy of PTH and BSAP to discriminate between low and non‐low turnover type. Two large bone biopsy studies characterized contemporary trends in prevalence rates of ROD turnover types and the diagnostic accuracy of PTH and BSAP for turnover.^3^, ^10^ In 630 dialysis patients, Malluche and colleagues^3^ reported that low turnover ROD was prevalent in the majority of patients (58%). Levels of PTH were lower in patients with low compared with high turnover, and total alkaline phosphatase did not differ between ROD turnover types. A second study of 492 patients on hemodialysis was led by a KDIGO consortium and assessed the diagnostic accuracy of PTH and BSAP for turnover type.^10^ Similar to Malluche and colleagues^3^ the prevalence of low turnover predominated (59%). PTH and BSAP insufficiently differentiated between low or high turnover to guide ROD treatment confidently: For PTH and BSAP, the AUC for discriminating low versus non‐low turnover was 0.701 and 0.757, respectively, and for discriminating high versus non‐high turnover ROD was 0.724 and 0.711, respectively. Combining PTH with BSAP did not improve accuracy for identifying either low or high turnover ROD. Sprague and colleagues^(10)^ also assessed diagnostic test characteristics for P1NP, which did not differ from those of PTH or BSAP. Among nondialysis CKD patients, diagnostic test characteristics of PTH, BSAP, P1NP, OCN, and TRAP‐5b for turnover type were similar to those reported for patients on dialysis.^11^, ^12^, ^51^, ^52^ Our investigation assessed diagnostic test characteristics for markers of CKD‐MBD [PTH, 25(OH)D, BSAP, FGF‐23], of bone formation (P1NP, OCN), and resorption (C‐telopeptide, TRAP‐5b) and of WNT signaling (SOST) for discrimination of ROD turnover type within the three bone compartments. We found differential discrimination of low turnover within trabecular, endocortical, and intracortical bone. Within trabecular bone, markers of CKD‐MBD and BTMs had moderate discrimination, and a biomarker panel including BSAP and CTx had excellent discrimination. Individually, these circulating markers had discrimination that was consistent with those of PTH and BSAP from the largest bone biopsy study to date (0.701 and 0.757, respectively).^3^, ^10^ However, it is noteworthy that the markers did not discriminate low turnover within cortical bone. In contrast, the miRNAs discriminated in cortical (both the endo‐ and intracortical compartments) bone. These findings may be consistent with the known differential effects of PTH on trabecular and cortical bone remodeling. Although the underlying mechanisms of anabolic and catabolic effects of PTH on trabecular and cortical bone, respectively, are unclear, the differences in discrimination of low turnover between compartments for the various biomarkers may be explained by these same molecular mechanisms.^53^, ^54^ Further research is needed to determine the mechanisms by which PTH modulates turnover in the bone compartments and miRNA expression.

Our data are the first to use a novel miRNA approach to identify novel noninvasive biomarkers of ROD turnover type. There is a growing body of literature on relationships between miRNAs and the skeleton.^14^, ^15^, ^16^, ^17^, ^18^, ^19^, ^21^, ^22^, ^23^, ^24^, ^25^, ^55^, ^56^ Dysregulation in levels of circulating miRNA expression has been noted in patients with osteoporosis^21^, ^22^, ^23^ and fractures.^24^, ^25^ Changes in levels of circulating miRNA caused by treatment with teriparatide and denosumab have been reported to correlate with changes in BTMs and BMD.^56^ However, Feurer and colleagues^55^ recently reported on relationships between 32 a priori selected miRNAs and fracture, BMD, and microarchitecture and BTMs in women with osteoporosis and healthy kidney function. They reported that miRNAs did not correlate with circulating BTMs and relationships between miRNAs and bone outcomes were negated by age.^55^ In CKD patients, levels of miRNAs and PTH have been correlated^26^; in cell culture, inorganic phosphate was shown to modulate osteoclastogenesis by miRNA‐233,^27^ but miRNAs have not been tested as biomarkers of turnover against the gold standard bone biopsy. We found that circulating miRNAs were not affected by kidney function, which is highly relevant to their utility across CKD grades. Similar to Feurer and colleagues,^55^ we did not find that miRNAs correlated with PTH, 25(OH)D, BSAP, or other markers of CKD‐MBD or bone turnover. This may reflect differences in their relationships with cellular processes and gene networks occurring at the bone tissue level. Indeed, our animal models suggest that levels of circulating miRNAs reflect miRNA expression in bone tissue and may represent a direct noninvasive marker of bone cell activity. In contrast, levels of calciotropic hormones, such as PTH, are regulated by phosphorus and calcium rather than bone cellular activity. Bone turnover markers reflect osteoblast and osteoclast activity, but OCN, P1NP monomer, and C‐telopeptide are cleared by the kidney and circulating levels may not accurately reflect bone cell activity, in particular, when renal function is impaired. We found that a panel of miRNAs more accurately discriminated low versus non‐low turnover ROD than a single miRNA: a finding that is consistent with data in other diseases such as hepatocellular cancer.^57^ These data need to be confirmed in future studies with larger cohorts of patients, with human bone tissue level confirmation of miRNA expression patterns, and with studies demonstrating that the miRNA profile changes in response to bone tissue level changes in turnover.

We conducted studies to quantify bone tissue expression levels of miRNAs in a rat model of ROD to confirm bone as a source of these miRNA. The mechanism of developing low turnover was either treatment of calcium in drinking water to reduce levels of PTH or the administration of zoledronic acid. Similar to circulating miRNA profiles in humans, bone tissue expression of the four miRNAs was lower in rats with low turnover induced by low PTH, and bone tissue expression of miRNA‐30c and 125 was lower in rats with low turnover, in the setting of high PTH, induced by zoledronic acid compared with bone from rats with high turnover. These results suggest that lower bone miRNA expression is reflecting the low turnover in CKD regardless of PTH levels.

Our investigation has limitations. This was a small cross‐sectional study of patients recruited at a single center. Although future work is needed in larger prospective cohorts to validate these data, our reported AUCs for PTH and BSAP are consistent with those reported in other studies of patients with CKD. Furthermore, data are needed to demonstrate that the miRNA profile changes in response to changes in turnover type, whether based on the natural history of ROD or caused by treatment effects. The miRNA panel that we identified had accurate discrimination for low versus non‐low turnover in cortical bone, which has been shown to be a critical bone compartment affected by ROD. This panel of miRNAs did not relate to turnover in trabecular bone and relationships between other miRNAs and turnover in trabecular bone need to be explored. Although our animal data suggest that bone tissue miRNA expression is reflected by bone turnover status, studies are needed to determine circulating miRNA in animals, the cell origin of these miRNAs (eg, osteoblast, osteocyte, osteoclast), and human bone tissue miRNA expression levels are needed.

In conclusion, we identified four circulating miRNA biomarkers that discriminated low from non‐low bone turnover ROD in cortical bone. Further research is needed to validate their diagnostic test characteristics, determine their responsiveness to the dynamic and complex clinical presentations of bone disease in patients with CKD, and identify other putative miRNA biomarkers of low and high turnover ROD and demonstrate that they inform clinical management.

## Disclosures

TN receives research support and is on the scientific advisory board of Amgen. PE receives research support, is a member of the speaker's bureau, and is on the scientific advisory board of Amgen. FM‐W is on the scientific advisory board and receives conference honoraria from Amgen, Sanofi, and Otsuka. SM is a scientific consultant for Amgen, and receives research support from Chugai and Keryx. NC, DM, DD, HZ, JD, MAA, JS, PD'H, and RM have nothing to disclose.

## Supporting information


**Supplemental Figure 1:** *Diagram of trabecular, endocortical and intracortical bone compartment segmentation*. The trabecular and endocortical envelopes include all interior bone surfaces in contact with the bone marrow space; the endocortical envelop is then defined as the bone surface lining the cortex. If segmentation of the inner boundary of cortex includes or straddles an open space, it is considered to be a bone marrow extension if the thickness of the trabecula separating the open space from the bone marrow cavity is ≤ radius of the open space; therefore, the open space is excluded from the inner boundary of the cortex and included as part of the trabecular envelope. The intracortical bone surface is referred to as the Haversian or osteonal canal surface and defined as the surface of cortical porosity where there are enlarged Haversian or osteonal canals ≥ 50 μm in diameter.
**Supplemental Figure 2:** Scatter plots between miRNA‐30b, 30c, 125b and 155 and kidney function. Patients on hemodialysis are indicated at the extreme left of the scatter plots. There was no relationships between the miRNAs and kidney function.
**Supplemental Figure 3:** Histomorphometric analysis for mineral apposition rate (a), mineralizing surface (b) and bone formation rate (c) of bone from CKD rats fed a calcium deficient or calcium containing diet, and rats given zoledronic acid and a calcium deficient diet. Data are shown as mean ± SD (*n* = 8–‐10 rats each group). *p < 0.05 CKD vs. CKD/Ca or CKD/Zol
