# Supplementary material for: A microRNA Approach to Discriminate Cortical Low Bone Turnover in Renal Osteodystrophy
Source: JBMR Plus. 2020 Mar 25;4(5):e10353. doi: 10.1002/jbm4.10353 (PMC7254487; doi:10.1002/jbm4.10353)
Supplement: Supplementary file 1 — Supplemental Figure 1: Diagram of trabecular, endocortical and intracortical bone compartment segmentation. The trabecular and endocortical envelopes include all interior bone surfaces in contact with the bone marrow space; the endocortical envelop is then defined as the bone surface lining the cortex. If segmentation of the inner boundary of cortex includes or straddles an open space, it is considered to be a bone marrow extension if the thickness of the trabecula separating the open space from the bone marrow cavity is ≤ radius of the open space; therefore, the open space is excluded from the inner boundary of the cortex and included as part of the trabecular envelope. The intracortical bone surface is referred to as the Haversian or osteonal canal surface and defined as the surface of cortical porosity where there are enlarged Haversian or osteonal canals ≥ 50 μm in diameter. Supplemental Figure 2: Scatter plots between miRNA‐30b, 30c, 125b and 155 and kidney function. Patients on hemodialysis are indicated at the extreme left of the scatter plots. There was no relationships between the miRNAs and kidney function. Supplemental Figure 3: Histomorphometric analysis for mineral apposition rate (a), mineralizing surface (b) and bone formation rate (c) of bone from CKD rats fed a calcium deficient or calcium containing diet, and rats given zoledronic acid and a calcium deficient diet. Data are shown as mean ± SD (n = 8–‐10 rats each group). *p < 0.05 CKD vs. CKD/Ca or CKD/Zol [file JBM4-4-e10353-s001.docx]

**Supplemental Data**

**Supplemental Figure 1**: *Diagram of trabecular, endocortical and intracortical bone compartment segmentation.* The trabecular and endocortical envelopes include all interior bone surfaces in contact with the bone marrow space; the endocortical envelop is then defined as the bone surface lining the cortex. If segmentation of the inner boundary of cortex includes or straddles an open space, it is considered to be a bone marrow extension if the thickness of the trabecula separating the open space from the bone marrow cavity is ≤ radius of the open space; therefore, the open space is excluded from the inner boundary of the cortex and included as part of the trabecular envelope. The intracortical bone surface is referred to as the Haversian or osteonal canal surface and defined as the surface of cortical porosity where there are enlarged Haversian or osteonal canals ≥ 50 µm in diameter.

**
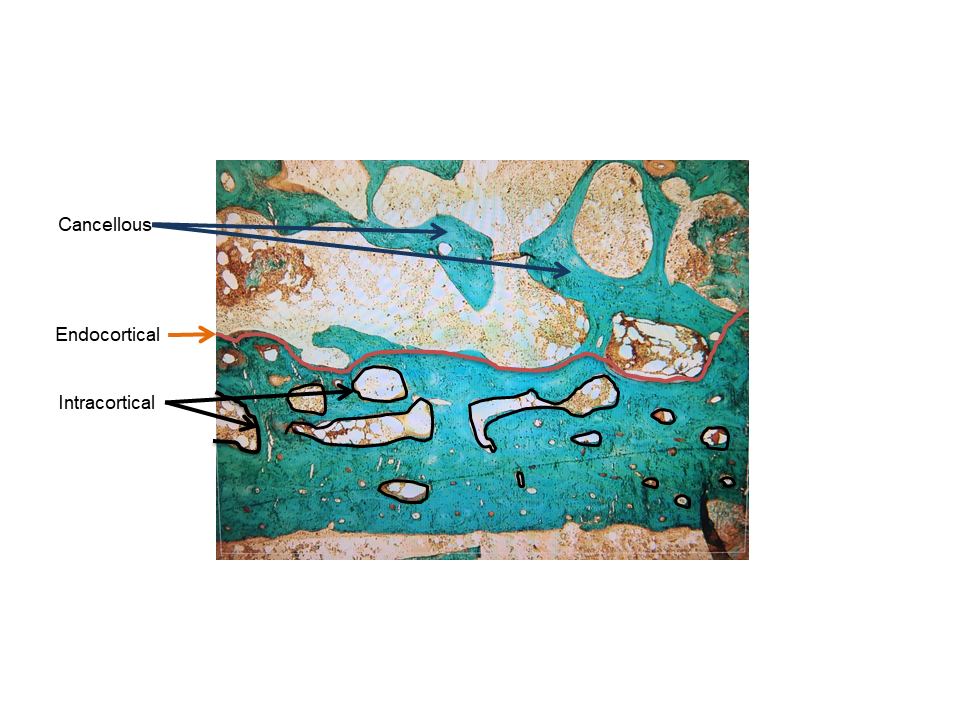
**

**Supplemental Figure 2:** Scatter plots between miRNA-30b, 30c, 125b and 155 and kidney function. Patients on hemodialysis are indicated at the extreme left of the scatter plots. There was no relationships between the miRNAs and kidney function.

**
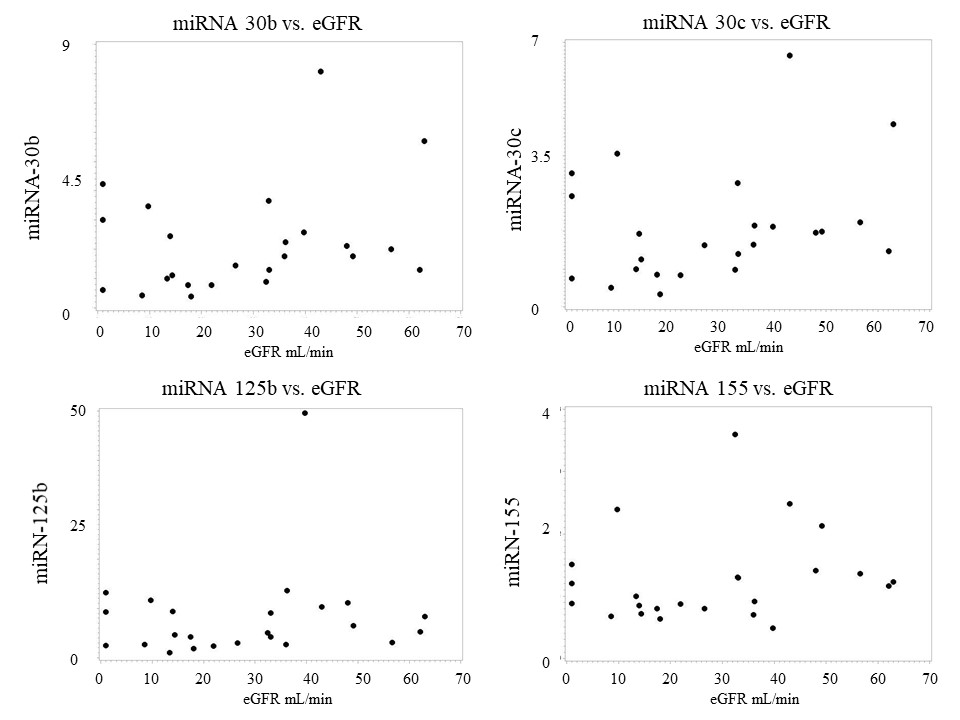
**

**Supplemental Figure 3:** Histomorphometric analysis for mineral apposition rate (a), mineralizing surface (b) and bone formation rate (c) of bone from CKD rats fed a calcium deficient or calcium containing diet, and rats given zoledronic acid and a calcium deficient diet. Data are shown as mean ± SD (n =8-10 rats each group). * p< 0.05 CKD vs. CKD/Ca or CKD/Zol

*

*

*

*

*

a.

b.

c.

*
